# Changing Management of Type B Aortic Dissections

**DOI:** 10.14797/mdcvj.1171

**Published:** 2023-03-07

**Authors:** John F. Eidt, Javier Vasquez

**Affiliations:** 1 Texas A&M College of Medicine, Bryan, Texas, US; 2 Baylor Scott and White Heart and Vascular Hospital, Dallas, Texas, US

**Keywords:** aortic dissection, Knickerbocker, Stablilise, Instead, stable, thoracic endograft, false lumen, malperfusion

## Abstract

The purpose of this manuscript is to review recent trends in the management of acute type B aortic dissection. Due to its efficacy and low morbidity, thoracic endografting has rapidly been adopted as the treatment of choice for most patients with malperfusion or rupture as a consequence of acute aortic dissection. This technology is increasingly applied to patients without rupture or malperfusion, so-called “uncomplicated” dissections, to reduce the incidence of late aneurysmal degeneration in the ungrafted segments of the thoracoabdominal aorta. A variety of techniques have been proposed, including intentional rupture of the dissection membrane to obliterate the false lumen as well as the candy-plug technique to eliminate retrograde flow in the false lumen.

## Introduction

Aortic dissection is the most common of the acute aortic syndromes. Despite contemporary advances in medical and surgical care, acute aortic dissection remains a highly lethal and morbid condition. It is estimated that up to a third of patients never reach the hospital.^1^ Of those surviving to reach definitive care, overall in-hospital mortality of type B aortic dissection (TBAD) approaches 15%.^2^ Recent epidemiological studies report a relatively stable incidence of acute aortic dissection between 3.5 and 6 per 100,000.^3,4,5^ Swedish researchers reported a significantly higher incidence of acute aortic dissection in men than women (9.1/100K vs 5.4/100K, *P* < .001).^1^ There appears to be an association with the increasing prevalence of hypertension in population-based studies.^2^

This article explores four significant trends in the management of type B aortic dissection: (1) adoption of a new classification system that resolves deficiencies of the DeBakey and Stanford systems; (2) recognition that thoracic endovascular aortic repair (TEVAR), the standard of care for treatment of complicated TBAD, fails to effectively prevent aneurysmal degeneration in the untreated aortic segments; (3) increasing use of TEVAR in uncomplicated TBAD despite the absence of definitive proof of efficacy; and (4) the emergence of a variety of techniques and devices designed to improve the long-term outcomes of TBAD.

## Anatomic Classification

**Figure 1:**
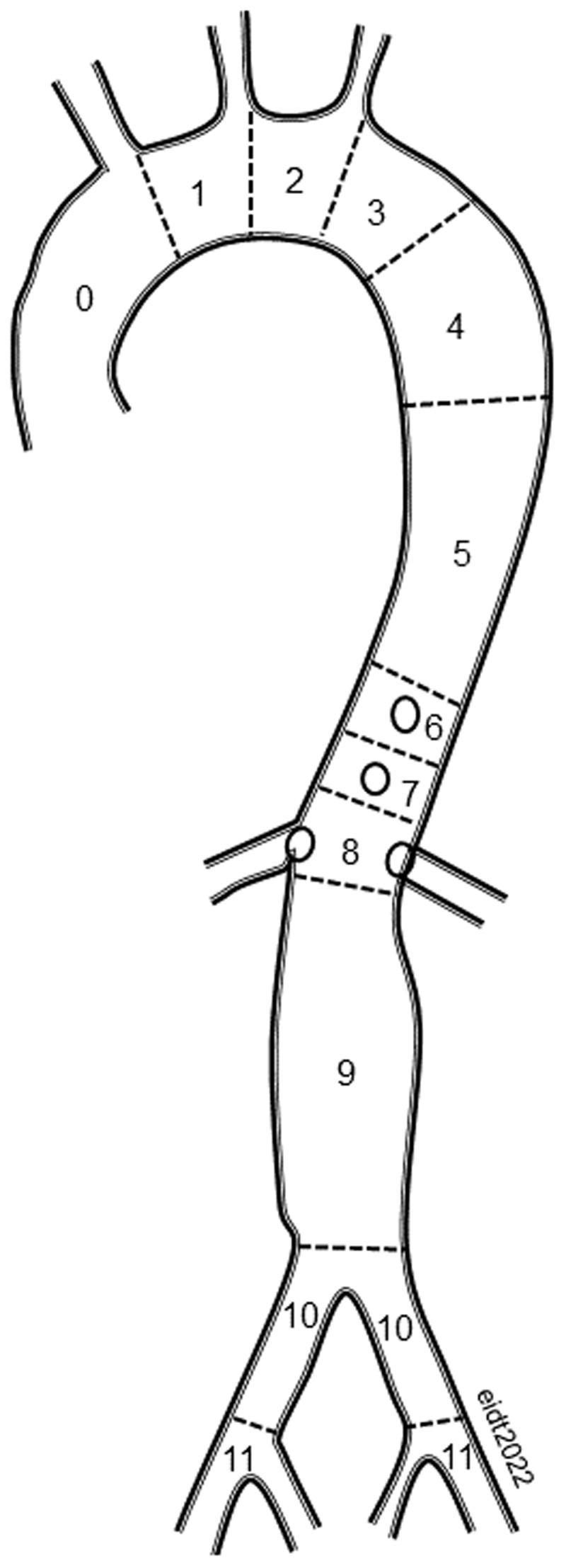
The thoracoabdominal aorta and the bifurcation are divided into 11 zones starting at the aortic valve and extending into the external iliac arteries. Acute dissections that involve the ascending aorta (zone 0) are designated type A. It is noteworthy that dissections involving zone 0 are predominantly intrapericardial, which can lead to the lethal triad of aortic insufficiency, pericardial tamponade, and coronary malperfusion. All dissections involving zones 1-11 are classified as type B (extrapericardial).

A number of classification schemes have been applied to aortic dissections, including the widely known DeBakey and Stanford systems. Both of these systems suffer from substantial deficiencies that led the Society for Vascular Surgery (SVS) and the Society of Thoracic Surgeons (STS) in 2020 to publish reporting standards that recommend the adoption of a new classification system based on the location of the primary entry tear and the longitudinal extent of the dissection.^3^ The aortoiliac arteries are divided into 11 zones beginning at the aortic valve and extending to the external iliac arteries (Figure 1). Type A aortic dissection (TAAD) describes a dissection in which the intimal tear is located in zone 0 of the ascending aorta.

In TBAD, the primary entry tear is in zones 1 through 11. The longitudinal extent of the dissection is designated with subscript zone numbers. Dissection includes the associated entities of intramural hematoma as well as partially or completely thrombosed false lumen. For example, a dissection with an entry tear in zone 3 and retrograde intramural hematoma encroaching on the left common carotid artery and extending into the left common iliac artery would be designated B_2-10_. A dissection with an entry tear in zone 0 extending to zone 4 would be designated A_4_ since the proximal extent is known to be zone 0. Finally, any dissection involving zone 0 but without an identifiable entry tear should be designated “I” for “indeterminate.”

The new system is based on recognition that dissections involving the intrapericardial portion of the aorta carry a lethal triumvirate of risks including aortic insufficiency, pericardial tamponade, and coronary malperfusion. As a result, surgery is typically recommended for all but the most moribund patients with TAAD because survival in the absence of surgical repair is uncommon. In contrast, medical management of uncomplicated TBAD has, until recently, been the standard of care.

### Time-Based Classification: Chronicity

SVS/STS reporting standards recommend the following classification of aortic dissection based on the chronicity of dissection: (1) hyperacute < 24 hours; (2) acute 1 to 14 days; (3) subacute 15 to 90 days; and (4) chronic > 90 days. These categories were chosen because they reflect changes in the maturation of the dissection membrane that impact the response to treatment. In 1958, Hirst et al. reported that 21% of patients with acute or hyperacute (TAAD and TBAD) aortic dissection died within 24 hours, 80% died within 4 weeks, and 93% died within 1 year.^6^ Recently, the Starnes group at the University of Washington reported that all-cause mortality exclusively for TBAD was 15% at 1 year, 24% at 3 years, and 57% at 10 years.^7^ The cumulative incidence of aorta-related mortality was 8.9% at 1 year, 16.5% at 3 years, and 27.2% at 10 years and that of non-aorta-related mortality was 2.7%, 7.2%, and 29%, respectively.^7^

## Acute Type B Dissection

### Classification of Acute TBAD (Complicated, Uncomplicated, and High-Risk)

Acute TBAD most commonly presents with the sudden onset of back pain, although chest, abdomen, and extremity pain also are reported.^2^ The most serious consequences of acute aortic dissection are rupture and malperfusion. Most patients with frank rupture of TBAD never reach the hospital, or they arrive in extremis and expire before definitive treatment. Patients with contained rupture or signs of malperfusion are classified as “complicated” TBAD, and most require urgent surgical treatment to avoid excessive morbidity and/or mortality (Table 1).^3^

**Table 1:** Features of complicated, uncomplicated, and high-risk acute type B aortic dissection.^3^


UNCOMPLICATED	COMPLICATED	HIGH RISK

		True lumen diameter > 22 mm
No high-risk features	Malperfusion	Total aortic diameter > 40 mm
No malperfusion	Rupture	Radiographic malperfusion
No rupture		Refractory pain Hemothorax Refractory hypertension Readmission


“Uncomplicated” TBAD dissections have no evidence of either frank or contained rupture and have no signs of end-organ malperfusion. Traditionally, these “uncomplicated” patients have been treated with optimal medical management, which entails “anti-impulse therapy” that employs strict blood pressure control as well as continuous monitoring for signs of end-organ ischemia or internal bleeding. Increasingly, these “uncomplicated” patients are treated with thoracic endovascular aortic repair (TEVAR) because of the recognition that the long-term success of medical management is suboptimal due to aneurysmal expansion of the false lumen.

The STS/SVS identifies a third group of patients with TBAD who are at particularly high risk for the development of complications.^3,4^ Radiographic predictors of adverse aortic outcomes include total aortic diameter > 40 mm, false lumen > 22 mm, primary entry tear on the inner curve of the aortic arch (as opposed to the outer curve), and evidence of subclinical malperfusion such as asymmetrical renal perfusion on computed tomography angiography (CTA). Hemothorax based on characteristic imaging findings or confirmed with needle aspiration is a particularly worrisome finding. Other high-risk clinical features include refractory hypertension, persistent chest pain, and/or the need for hospital readmission typically due to recurrent pain and inadequate outpatient control of blood pressure.

### Management of Rupture in Complicated Acute TBAD

There is little debate that emergency treatment of ruptured TBAD is appropriate in all but the most moribund patients. Patients should be taken immediately to a suitable procedure room with both endovascular and open surgical resources for resuscitation and treatment. Chest tubes should generally be discouraged prior to TEVAR for fear of inducing hemodynamic instability from sudden chest decompression. Permissive hypotension is recommended to avoid disruption of a contained rupture by exuberant fluid resuscitation. Endovascular coverage of the bleeding site with a thoracic endograft has emerged as the standard of care. Adjunctive obliteration of the retrograde false lumen filling from distal re-entry tears (eg, “candy plug,” coil embolization, or Knickerbocker technique) may be necessary to achieve complete hemostasis after proximal TEVAR.^8,9,10^ Thankfully, unless no endovascular option exists, open surgical treatment of ruptured TBAD has all but vanished due to poor operative outcomes. In-hospital mortality in cases of TBAD with rupture ranges from 25% to 50%.

### Management of End-Organ Malperfusion in Complicated Acute TBAD

Acute TBAD can result in obstruction to blood flow in any organ supplied by the involved segment of the aorta and its branches. For patients with malperfusion, the success of endovascular management is largely dependent on the location of the primary entry tear, the longitudinal extent of the dissection, and the impact of the dissection on branch vessel patency.^11^ In the most common scenario, the primary entry tear is located in zone 3 with variable propagation of the dissection both proximally (retrograde) and distally (antegrade). TEVAR is typically effective at achieving the dual objectives of covering the proximal entry tear and restoring, or at least improving, true lumen blood flow. If branch vessel obstruction persists after TEVAR, percutaneous stenting of branches to assure communication between the true lumen and the end organ is usually effective at restoring blood flow. By reducing the inflow to the false lumen at the primary entry tear, TEVAR is expected to reduce the pressurization of the false lumen with subsequent improvement in true lumen flow. There is a complex interplay between the size and location of the primary entry tear and the location, number, and size of the re-entry tears. The combination of a large proximal entry tear and limited (or no) exit tears has been associated with high false lumen pressurization and the risk of aneurysmal enlargement.

Traditionally, branch vessel obstruction has been classified as either dynamic or static. Dynamic obstruction is the result of the variable pressurization of the false lumen during the cardiac cycle. It can be visualized with intravascular ultrasound (IVUS), which demonstrates the remarkable mobility of the dissection membrane. Static obstruction of branch vessels results from constant compression or thrombosis of the true lumen. Dynamic obstruction is estimated to be the dominant mechanism of ischemia in approximately 4 of 5 patients with malperfusion.^11^

If endovascular options are unavailable to treat malperfusion due to the absence of appropriate resources or personnel, a number of open surgical options may be enlisted. Open aortic septectomy via a left flank retroperitoneal exposure can effectively restore visceral and renal perfusion. Extremity ischemia can be treated with extra-anatomic bypass if endovascular options have failed or are unavailable.

## Trends and Outcomes in Tbad

Over the past 20 years since the widespread use of TEVAR, the following trends in management of acute TBAD have been observed: increasing use of TEVAR in uncomplicated patients, decreasing open surgery, and decreasing reliance on optimal medical management alone from more than 75% to approximately 50%.^12^ These trends are based on the observation that TEVAR in combination with contemporary endovascular adjunctive techniques is remarkably effective in the treatment of malperfusion and rupture in the setting of acute TBAD.

Approximately one-third of cases of acute TBAD are complicated. Overall, in-hospital mortality with acute TBAD is 12% to 14% with a three-fold increase in mortality in complicated/high risk patients.^2,12,13^ A number of predictors of mortality have been identified (Table 2). Mesenteric ischemia is a particularly ominous finding, with a nine-fold increased risk of death compared to patients with normal visceral blood flow.

**Table 2:** Predictors of mortality in acute type B dissection.^2^


PREDICTORS	MORTALITY ODDS RATIO

Increasing age per decade	1.3

Female sex	1.4

Extremity ischemia	3.0

Periaortic hematoma	3.0

Aortic diameter > 5.5 cm	3.0

Acute renal failure	3.6

Hypotension/shock	6.4

Mesenteric ischemia	9.0


### Long-Term Outcomes of Acute TBAD

A seminal report from the International Registry of Acute Aortic Dissection (IRAD) provides important background data for evaluation of long-term outcomes after acute TBAD treated with TEVAR (n = 276) versus optimal medical management (OMT) (n = 853).^14^ In-hospital mortality was similar in patients managed with TEVAR versus OMT (10.9% vs 8.7%, *P* = .273). One-year mortality also was similar in both groups (8.1% TEVAR vs 9.8% OMT, *P* = .604). Aortic enlargement was observed in 62.7% of patients after TEVAR and 73.3% after OMT based on 5-year Kaplan-Meier estimates.^14^ The long-term effectiveness of endovascular treatment of acute TBAD with TEVAR is largely determined by both the longitudinal extent and the chronicity of the dissection. While malperfusion is a problem predominantly confined to the hyperacute and acute phases (ie, up to 14 days), it has been observed that remodeling (true lumen expansion and false lumen regression) of the aorta after TEVAR occurs almost exclusively in the segment that is covered by the endograft.^15,16,17,18,19^ In addition, temporal changes in the structure and compliance of the dissection membrane have an important impact on long-term success.

Aortic remodeling after TEVAR is a continuous process.^20^ Acute dissections remodel more rapidly and more often than chronic dissections,^21^ and remodeling is greater in more proximal aortic segments.^18^ For dissections confined to zones 2 through 5 (from the left common carotid to the celiac artery), simple TEVAR with or without left subclavian artery revascularization is usually effective at achieving both short- and long-term success as measured by complete false lumen remodeling and avoidance of additional procedures.^22,23^ For acute TBAD involving zone 1, either endovascular revascularization or extra-anatomic surgical debranching of the left common carotid and left subclavian arteries can achieve equally durable long-term results. But in patients managed medically or in TEVAR patients with dissection involving the visceral aorta and the aortic bifurcation (zones 6-11), the long-term outcomes of acute and subacute TBAD are dominated by the inexorable enlargement of the untreated aortic segments. At least one-third of survivors of acute TBAD will require subsequent intervention of the untreated aortic segment, mostly as a result of pressurization of the false lumen that causes total aortic diameter expansion.

While TEVAR for chronic dissection with progressive false lumen enlargement has been associated with low perioperative risk, Mani et al. reported that total aortic diameter was reduced in only 50% of patients following TEVAR for chronic aortic dissection.^15^ Others have reported similar disappointing mid- and long-term outcomes following conventional TEVAR.^24,25^ Thus, substantial efforts are underway to improve the long-term outcomes by procedural modifications to the endovascular treatment of acute TBAD. At least three strategies have developed with sufficient momentum to warrant review here: (1) techniques designed to enlarge the true lumen and aggressively obliterate the false lumen, (2) techniques to induce false lumen thrombosis and advance remodeling, and (3) improvements in endograft design to reduce long-term adverse events.

### OMT vs TEVAR in acute uncomplicated TBAD

It is widely believed that thrombosis of the false lumen and subsequent remodeling of dissected aorta is likely associated with improved survival.^15,23,26,27^ While OMT is effective in the near-term, it has not prevented aneurysmal degeneration of the dissected aorta. The INSTEAD trial randomly assigned patients with uncomplicated, subacute (> 14 d) TBAD to OMT (n = 68) or OMT and TEVAR (n = 72).^28^ Initial results showed no difference in all-cause mortality at 2 years, but 5-year data confirmed improved survival and aortic remodeling in the TEVAR group.^29^ It should be noted that the INSTEAD trial specifically excluded patients < 14 days and may not reflect outcomes in hyperacute or acute-phase patients. The ADSORB trial randomly assigned patients with acute (< 14 days) TBAD to OMT (n = 31) or OMT plus TEVAR (n = 30). One-year results showed improved remodeling of the thoracic aorta covered by an endograft,^30^ and long-term results are pending. Of note, perioperative paraplegia, stroke, and death were not observed in ADSORB.^30^ One major stroke and two cases of spinal cord injury (SCI) were reported in the INSTEAD trial.^28^

These data support the concept of employing TEVAR in selected uncomplicated TBAD to reduce the incidence of late aneurysmal change. For TEVAR to play a role in these patients, it must be accomplished with minimal morbidity, which from a practical standpoint means avoiding stroke and SCI. There are insufficient data to support the widespread adoption of TEVAR for uncomplicated TBAD at this time. A large randomized multicenter trial would be required to definitively answer this question.

## Techniques to Prevent Aneurysmal Degeneration of Tbad

### Knickerbocker

**Figure 2:**
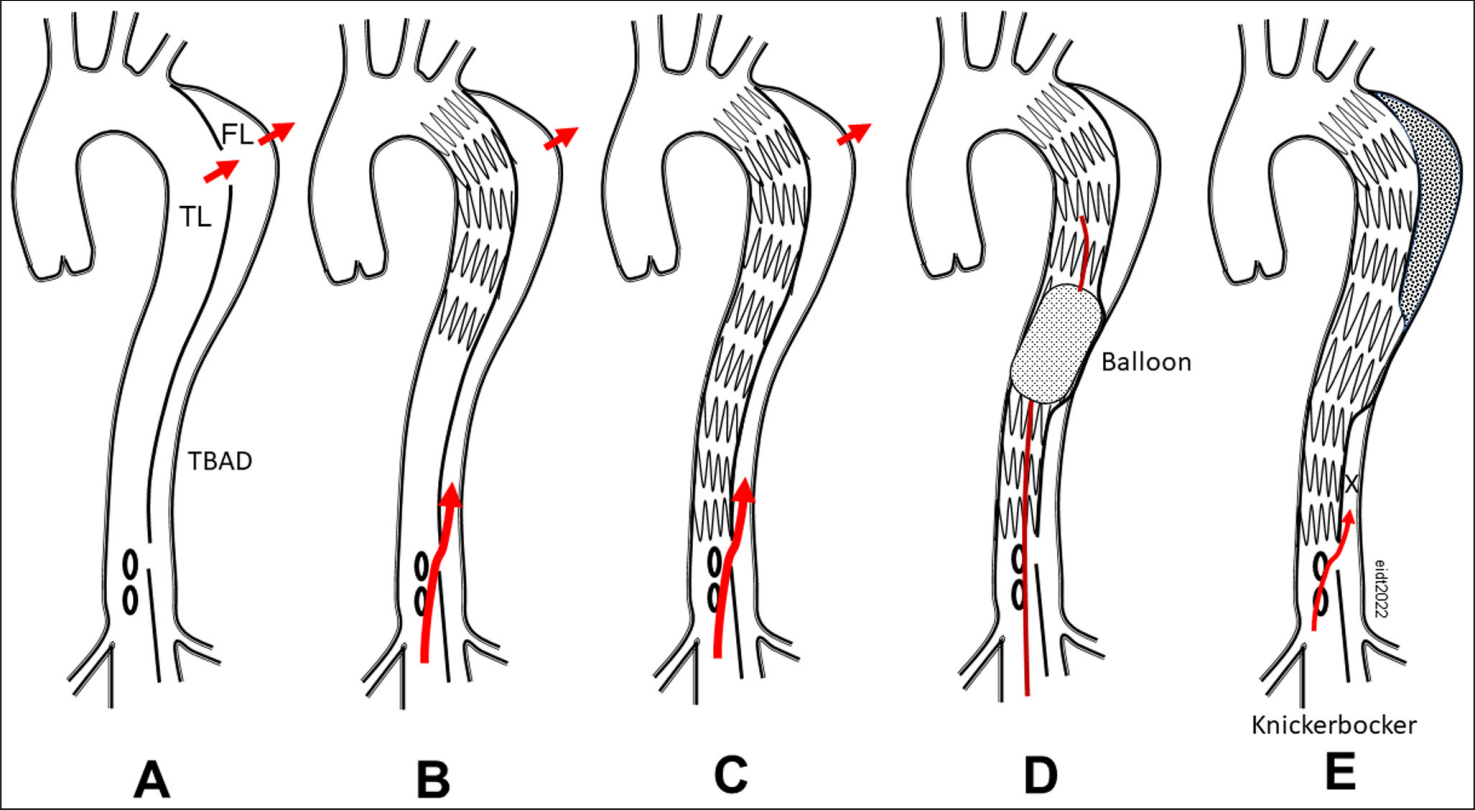
Illustration of the Knickerbocker technique. **(A)** Type B aortic dissection with a large proximal entry tear leading to bleeding in the chest. **(B)** After TEVAR, the proximal entry tear is covered but distal false lumen backflow persists. **(C)** Despite covering the entire length of the descending thoracic aorta, there is persistent bleeding via false lumen filling. **(D)** Inflating a compliant balloon within the endograft intentionally ruptures the dissection membrane. **(E)** The expanded endograft prevents backflow into the false lumen aneurysm and eliminates the source of intrathoracic hemorrhage (X). TEVAR: thoracic endovascular aortic repair; TL: true lumen; FL: false lumen; TBAD: type B aortic dissection

The “knickerbocker” technique was first described in 2014 in three patients with subacute or chronic aortic dissections (Figure 2).^31^ All three patients had either rapid expansion or frank rupture from aneurysmal enlargement of the false lumen. Following placement of a thoracic endograft that was oversized 10% to 15% larger than the total aortic diameter, persistent hemorrhage occurred due to retrograde flow in the false lumen from a re-entry tear. Given the urgency, the authors elected to rupture the dissection membrane with a compliant balloon in the midportion of the endograft and thereby expand the endograft to obliterate the false lumen and prevent retrograde exsanguination. The endovascular technique was designed to mimic a similar open transsternal procedure reported by Rosselli et al. or a subxiphoid technique by Konings.^32,33^ The authors eventually contributed to the design of a commercial thoracic endograft with a double-tapered tubular design and a bulbous middle segment (similar to the shape of knickerbocker pants) that could be intentionally expanded to obliterate the false lumen and prevent back flow.^34^ The device has not gained widespread use but established the feasibility of balloon membrane rupture to eliminate the false lumen, a technique that would be subsequently modified by others to achieve more complete treatment of TBAD using the STABILISE technique.^35,36,37,38,39,40,41,42,43,44,45,46,47^

### Petticoat

**Figure 3:**
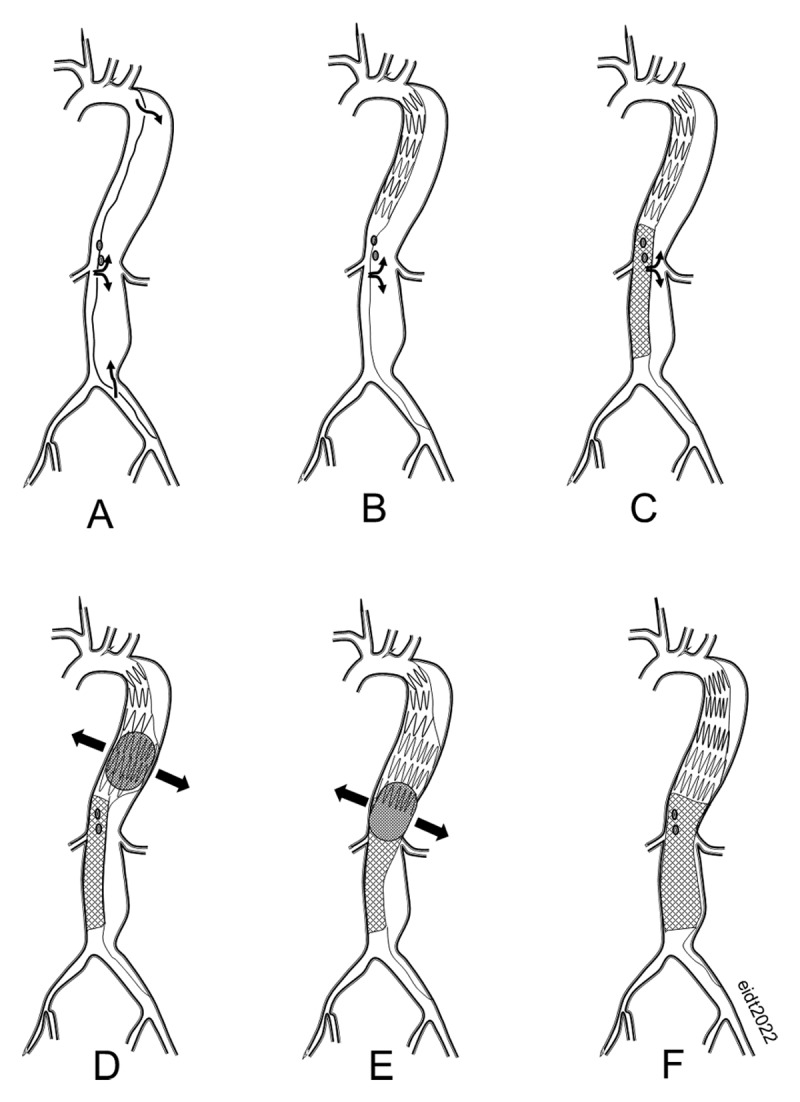
**(A)** Acute type B aortic dissection. **(B)** Following proximal thoracic endografting, there is persistent retrograde flow in the false lumen. **(C)** Bare metal PETTICOAT self-expanding stent improves true lumen blood flow but does not prevent false lumen filling. **(D)** Intentional rupture of the dissection membrane with a compliant balloon results in obliteration of the false lumen. **(E)** The balloon is sequentially inflated throughout the entire thoracic and abdominal aorta. **(F)** The false lumen is completely eliminated by intentional rupture of the dissection membrane.

An alternative strategy to reduce the incidence of late treatment failures following TEVAR for TBAD relies on a composite device with a covered proximal component and bare metal distal component that is extended through the visceral segment (zones 6-8) to expand the true lumen and induce total aortic remodeling. Nienaber introduced the term “provisional extension to induce complete attachment after stent-graft placement in type B aortic dissection” (PETTICOAT) in 2006 to describe the use of bare metal self-expanding stents in the visceral aortic segment in 12 patients with persistent malperfusion despite proximal aortic endografting (Figure 3).^48^ The addition of the bare metal stent was uniformly effective in relieving acute malperfusion.

Since its introduction, the technique has been used with increasing enthusiasm with the goal of improving late aortic remodeling in the visceral aortic segment. One of the largest trials (STABLE) evaluated the Zenith Dissection Endovascular System (Cook, Inc.), consisting of a conventional proximal endograft combined with bare metal Z-stents extending through the abdominal aorta.^49^ This prospective, single-arm, multicenter study enrolled 86 patients at sites in Europe, Australia, and the United States from 2007 to 2012. All patients had acute or subacute dissections (< 90 days), and 30-day all-cause mortality was 4.7%. At 5 years, freedom from all-cause mortality was 75% and freedom from aorta-related mortality was 85%.^50^ Complete thrombosis of the false lumen in the thoracic aorta was observed in approximately 70% (30/44) of patients but in < 10% (4/46) in the abdominal aorta.^50^ Approximately 30% required reintervention within 5 years. While these results represent incremental improvement over standard TEVAR without visceral aortic bare metal stenting, it was disappointing that remodeling in the uncovered aortic segments was not more impressive.

Despite dozens of publications reporting the results of the PETTICOAT technique, there are no head-to-head or randomized comparisons between conventional proximal TEVAR and TEVAR plus visceral bare-metal stenting. Results of the PETTICOAT trials have been mixed. For example, Nienaber et al. reported improved remodeling of the abdominal aorta at the level of the celiac artery in patients treated with TEVAR plus E-XL bare stent (JOTEC) compared with TEVAR-only propensity-matched controls (complete false lumen thrombosis 54% versus 18%, *P* = .004).^51^

In contrast, Mascia et al. reported that a PETTICOAT using the Zenith bare stent system does not prevent long-term aneurysmal degeneration.^52^ Furthermore, there have been occasional reports of complications related to the bare stent, including stent migration, stent fracture, and misalignment.^53,54,55^ A Cochrane review of the long-term outcomes of the PETTICOAT was abandoned because the absence of randomized trials precluded definitive assessment of the technique’s long-term utility.^56^ A meta-analysis by Qui et al. that included 914 patients in eight observational studies reported no difference in overall aorta-related mortality or complete false lumen thrombosis in the thoracic or abdominal segments. The bare stent group did have fewer stent-graft–induced new entry tears and less frequent reintervention.^57^

In summary, it appears that the extension of a bare metal stent in the abdominal aorta following proximal endografting is clearly beneficial in the acute setting because it improves visceral perfusion. The benefit of the PETTICOAT technique for reducing long-term adverse aortic events remains unproven.

### STABILISE Technique

In an effort to reduce retrograde false lumen filling after proximal thoracic TEVAR, Hofferberth et al. in 2014 introduced the STABILISE concept (Stent-assisted Balloon-Induced Intimal Disruption and Relamination in Aortic Dissection Repair).^47^ In this technique, a proximal endograft is placed in the thoracic aorta to cover the primary entry tear and a bare-metal “dissection” stent is extended through the visceral segment into the infrarenal aorta. In a deviation from the usual PETTICOAT technique, which depends entirely on the passive outward force of the self-expanding bare stent for compression of the false lumen, a compliant balloon is used to rupture the dissection membrane beginning in the thoracic endograft and extending to the distal extent of the bare stent.^47^ This technique effectively creates a single aorta channel and eliminates the false lumen. The chief drawback of this technique is fear of frank aortic rupture. In a search of all reported cases in the literature, there is only a single example of contained rupture of the infrarenal aorta, which was salvaged with a conventional EVAR device.^58^

The chief benefit of the STABILISE technique appears to be markedly improved remodeling of the abdominal aorta. Zhong et al. reported complete remodeling of the thoracic aorta in 100% (11/11) and of the abdominal aorta in 83% (9/11), which far exceeds historical controls.^58^ Others have reported similar results in both the acute and chronic setting using the STABILISE teichnique.^35,37,39,43,59,60^ Some have recommended more aggressive use of distal stenting and endografting in the infrarenal and iliac segments to cover all identifiable reentry tears. These reports suggest improved remodeling of the abdominal aortoiliac segment at the potential expense of increased SCI.^61,62,63^

### False Lumen Embolization

An alternative strategy to reduce false lumen enlargement long-term relies on a variety of techniques to induce thrombosis of the false lumen. In the original “candy plug” technique, a modified endograft component (eg, GORE EXCLUDER cuff) was released in the false lumen adjacent to a proximal true-lumen TEVAR with the goal of occluding flow in the false lumen.^10^ A second-generation device was produced commercially exclusively for this purpose.^9^ Pellenc et al. reported satisfactory results of false lumen embolization with Amplatzer plugs and conventional coils in 27 patients with chronic TBAD.^64^ Thrombosis of the false lumen was observed in 22/27 patients, including two cases of spinal cord ischemia despite spinal drainage. Others have reported the use of patent foramen ovale or atrial septal defect occluders, coils, and glue in addition to endovascular stenting of the true lumen with satisfactory short-term results.^65^

### Exercise Following TEVAR

As with other surgical conditions, increasing emphasis is placed on patient-centered outcomes. One important consideration in patients after treatment for TBAD is guidance regarding appropriate activity. While activities that lead to sudden spikes in blood pressure clearly should be avoided (eg, bench press with closed glottis), evidence is scarce for guiding clinicians in making rational recommendations. A recent survey of IRAD members favors aerobic activities to maintain overall cardiovascular fitness while avoiding highly stressful ballistic training that could be hazardous.^66^

### Cerebrospinal Fluid Drainage

Cerebrospinal fluid drainage (CSFD) during and after procedures that interfere with spinal cord blood flow has been widely used for 40 years to reduce the incidence of SCI. During the last several years, it has become apparent that the low incidence of SCI after TEVAR (< 5%) and the known risks associated with CSFD (eg, epidural hematoma, subarachnoid hemorrhage, malfunction, and infection) favor therapeutic CSFD over prophylactic drainage in most cases.^67^ Permissive hypertension may have a more beneficial impact on relieving postoperative paraparesis than prophylactic CSFD.^67^

## Summary

TEVAR is the standard of care for the treatment of acute complicated TBAD and selected patients with high-risk features. TEVAR effectively manages both rupture and malperfusion in most patients with acute TBAD without the need for secondary intervention. TEVAR is being employed more frequently in uncomplicated TBAD with the caveat that periprocedural risks of paraplegia and stroke must approach zero to justify this “prophylactic” strategy. The long-term outcomes from proximal endografting alone have been disappointing due to continued aneurysmal expansion in the untreated aortic segment occurring in 30% to 50% of patients within 5 years. A variety of other strategies have been explored including the PETTICOAT and STABILISE techniques to reduce the need for secondary interventions to prevent aneurysm formation.

## Key Points

- There is a new classification system that resolves deficiencies of the DeBakey and Stanford systems.
- It is recognized that thoracic endovascular aortic repair (TEVAR), the standard of care for treatment of complicated type B aortic dissection (TBAD), fails to effectively prevent aneurysmal degeneration in the untreated aortic segments.
- There is increasing use of TEVAR in uncomplicated TBAD despite the absence of definitive proof of efficacy.
- A variety of techniques and devices have emerged to improve the long-term outcomes of TBAD.
